# Supplementary material for: Climate induced stress and mortality in vervet monkeys
Source: R Soc Open Sci. 2019 Nov 13;6(11):191078. doi: 10.1098/rsos.191078 (PMC6894595; doi:10.1098/rsos.191078)
Supplement: Supplementary material [file rsos191078supp1.docx]

*Climate induced stress and mortality in vervet monkeys*

Christopher Young^a,b,c^, Tyler R. Bonnell^c^, Leslie R. Brown^b^, Marcus J. Dostie^c,d^, Andre Ganswindt^a^, Stefan Kienzle^b,d^, Richard McFarland^e,f^,_,_ S. Peter Henzi^b,c^ and Louise Barrett^b,c^

^a^Endocrine Research Laboratory, Mammal Research Institute, Faculty of Natural and Agricultural Science, University of Pretoria, Pretoria, Republic of South Africa, ^b^Applied Behavioural Ecology and Ecosystems Research Unit, University of South Africa, Pretoria, Republic of South Africa. ^c^Department of Psychology, University of Lethbridge, Alberta, Canada. ^d^Department of Geography, University of Lethbridge, Alberta, Canada. ^e^Department of Anthropology, University of Wisconsin-Madison, Madison, United States. ^f^Brain Function Research Group, School of Physiology, University of the Witwatersrand, Republic of South Africa.

**Supplementary material**

Table S1: Coefficient estimates for model 1_temp+water_ examining the influence of social and environmental factors on fGCM concentrations. Shown are the estimate of the posterior means, standard error of the estimate of the posterior means and the 95% credible intervals (CI). Given are estimates on the main effects and the residual variation between individuals. Bold highlighting indicates that the estimate is greater than ± 2 x the standard error and the majority of the 95% CI is either positive or negative.

| Factor | Estimateof posterior mean | Estimate Error | Lower 95% CI | Upper 95% CI |
| --- | --- | --- | --- | --- |
| *Main effects:* | | | | |
| Intercept | 3.95 | 0.09 | 3.79 | 4.12 |
| δ-intercept | 0.55 | 0.02 | 0.50 | 0.60 |
| Group: PT vs. RST | -0.08 | 0.09 | -0.27 | 0.10 |
| Group: PT vs. RBM | 0.05 | 0.11 | -0.16 | 0.26 |
| Group: RST vs. RBM | 0.13 | 0.10 | -0.05 | 0.33 |
| Standardized rank | -0.05 | 0.09 | -0.24 | 0.13 |
| Sex: Male | 0.07 | 0.09 | -0.1 | 0.24 |
| Days without water | 0.09 | 0.07 | -0.05 | 0.23 |
| Daily average temperature | 0.11 | 0.07 | -0.02 | 0.24 |
| **Interaction of days without water and daily average temperature** | **-0.21** | **0.15** | **-0.51** | **0.09** |
| *Individual residual variance:* | | | | |
| **Intercept** | **0.15** | **0.07** | **0.02** | **0.28** |
| **Standardized rank** | **0.30** | **0.14** | **0.03** | **0.59** |
| Days without water | 0.11 | 0.08 | 0.00 | 0.29 |
| Daily average temperature | 0.10 | 0.07 | 0.00 | 0.21 |
| Interaction of days without water and daily average temperature | 0.21 | 0.16 | 0.01 | 0.59 |

Table S2: Showing the output of the multinomial behavioural model of four categories of behaviour (moving, resting, foraging and social). Moving was set as the reference category. We included time period (cold-wet, hot-wet, hot-dry and cold-dry), sex (reference: male) and standardized dominance rank as fixed effects. Shown are the posterior means, standard deviation and the lower and upper 95% credibility intervals of the fixed effects.

| Variable | Behaviours compared | Mean | Standard deviation | Lower 95% CI | Upper 95% CI |
| --- | --- | --- | --- | --- | --- |
| Cold-wet | Social vs. moving | 0.88 | 0.06 | 0.76 | 1.01 |
|  | Resting vs. moving | 0.42 | 0.05 | 0.33 | 0.52 |
|  | Feeding vs. moving | 0.59 | 0.05 | 0.49 | 0.68 |
| Hot-wet | Social vs. moving | 0.58 | 0.05 | 0.48 | 0.67 |
|  | Resting vs. moving | 0.59 | 0.03 | 0.53 | 0.65 |
|  | Feeding vs. moving | 0.25 | 0.03 | 0.19 | 0.32 |
| Hot-dry | Social vs. moving | 0.66 | 0.05 | 0.5 | 0.75 |
|  | Resting vs. moving | 0.89 | 0.03 | 0.83 | 0.94 |
|  | Feeding vs. moving | 0.26 | 0.03 | 0.20 | 0.32 |
| Cold-dry | Social vs. moving | -1.38 | 0.21 | -1.72 | -0.83 |
|  | Resting vs. moving | -0.22 | 0.07 | -0.33 | -0.11 |
|  | Feeding vs. moving | -0.23 | 0.22 | -0.67 | 0.28 |
| Sex: Male | Social vs. moving | -0.85 | 0.10 | -1.05 | -0.66 |
|  | Resting vs. moving | 0.16 | 0.05 | 0.05 | 0.25 |
|  | Feeding vs. moving | -0.11 | 0.07 | -0.27 | 0.03 |
| Standardized dominance rank | Social vs. moving | 0.04 | 0.08 | -0.11 | 0.19 |
|  | Resting vs. moving | -0.01 | 0.04 | -0.09 | 0.07 |
|  | Feeding vs. moving | 0.09 | 0.05 | -0.01 | 0.18 |
